# Pm21 CC domain activity modulated by intramolecular interactions is implicated in cell death and disease resistance

**DOI:** 10.1111/mpp.12943

**Published:** 2020-05-18

**Authors:** Anli Gao, Minjie Hu, Yifei Gong, Ruixiang Dong, Yuan Jiang, Shanying Zhu, Jian Ji, Dale Zhang, Suoping Li, Huagang He

**Affiliations:** ^1^ School of Life Sciences Henan University Kaifeng China; ^2^ School of Food and Biological Engineering Jiangsu University Zhenjiang China

**Keywords:** CC domain, cell death, disease resistance, intramolecular interaction, Pm21

## Abstract

Nucleotide‐binding (NB) leucine‐rich repeat (LRR) receptors (NLRs) provide resistance against several plant pathogens. We previously cloned the wheat powdery mildew resistance gene *Pm21*, which encodes a coiled‐coil (CC) NLR that confers broad‐spectrum resistance against *Blumeria graminis* f. sp*. tritici*. Here, we report comprehensive biochemical and functional analyses of Pm21 CC domain in *Nicotiana benthamiana*. Transient overexpression assay suggested that only the extended CC (eCC, amino acid residues 1–159) domain has cell‐death‐inducing activity, whereas the CC‐containing truncations, including CC‐NB and CC‐NB‐LRR, do not induce cell‐death responses. Coimmunoprecipitation (Co‐IP) assay showed that the eCC domain self‐associates and interacts with the NB and LRR domains in planta. These results imply that the activity of the eCC domain is inhibited by the intramolecular interactions of different domains in the absence of pathogens. We found that the LRR domain plays a crucial role in D491V‐mediated full‐length (FL) Pm21 autoactivation. Some mutations in the CC domain leading to the loss of *Pm21* resistance to powdery mildew impaired the CC activity of cell‐death induction. Two mutations (R73Q and E80K) interfered with D491V‐mediated Pm21 autoactivation without affecting the cell‐death‐inducing activity of the eCC domain. Notably, some susceptible mutants harbouring mutations in the CC domain still exhibited cell‐death‐inducing activity. Taken together, these results implicate the CC domain of Pm21 in cell‐death signalling and disease‐resistance signalling, which are potentially independent of each other.

## Graphical abstract

Pm21 coiled‐coil is the cell‐death and disease‐resistance signalling domain whose activity is regulated by intramolecular interactions of different domains.

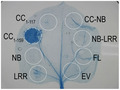

## 1 INTRODUCTION

Plants prevent pathogen invasion via a two‐layered immune system (Dodds and Rathjen, 2010; Dangl *et al.*, 2013). The first layer is pattern‐triggered immunity (PTI) governed by membrane‐localized pattern recognition receptors (PRRs). PTI is the basal immune response of plants against infections by pathogens (Couto and Zipfel, 2016; Tang *et al.*, 2017). Once pathogens suppress PTI and enter host cells, plants employ the second layer of the immune system to combat pathogens. In this branch of the immune system, intracellular nucleotide‐binding leucine‐rich repeat receptors (NLRs) directly or indirectly recognize pathogen effectors that are delivered into the cytosol of plant cells (Cui *et al.*, 2015; Zhang *et al.*, 2017). This defence system, governed by NLR, is termed effector‐triggered immunity (ETI) and triggers the death of cells around pathogen infection sites, which is believed to limit the spread of pathogens (Dangl *et al.*, 2013; Jones *et al.*, 2016).

NLR proteins contain variable N‐terminal domains, central nucleotide‐binding (NB) domains, and C‐terminal leucine‐rich repeat (LRR) domains (Cesari, 2018). Depending on the structure of their N terminal, NLRs are broadly classified into the following two types: one possessing a Toll‐interleukin 1 receptor (TIR) domain (named TIR‐NLR) and the other forming a coiled‐coil (CC) domain (named CC‐NLR) (Cui *et al.*, 2015). The central NB domain, highly conserved in all NLRs, is believed to function as a molecular switch of activity regulation, with ADP‐ and ATP‐bound forms defining the “off” and “on” state of NLRs, respectively. (Takken *et al.*, 2006; Williams *et al.*, 2011; Bernoux *et al.*, 2016). In support of this perspective, the crystal structure of ZAR1 shows that on effector perception, the NB domain of ZAR1 changes conformation and releases ADP to form an intermediate state (Wang *et al.*, 2019a). The LRR domain, which is the most variable region in the NLR proteins, is implicated in the perception of pathogen effectors and NLR autoinhibition (Dodds *et al.*, 2001; Deslandes *et al.*, 2003; Wang *et al.*, 2007; Krasileva *et al.*, 2010; Ravensdale *et al.*, 2012). A recent study suggested that the LRR domain of ZAR1 plays an important role in interaction with the decoy RKS1, while also helping to maintain ZAR1 in an autoinhibition state through intramolecular interactions (Wang *et al.*, 2019a). Both the CC and TIR domains have been shown to function as signalling components in plant NLRs (Swiderski *et al.*, 2009; Bernoux *et al.*, 2011; Baudin *et al.*, 2017; Wang *et al.*, 2018; Zhai *et al.*, 2019). Indeed, the CC or TIR domains of numerous NLR proteins can trigger cell death when transiently expressed in *Nicotiana benthamiana* (Bernoux *et al.*, 2011; Bai *et al.*, 2012; Wang *et al.*, 2015; Cesari *et al.*, 2016; Hamel *et al.*, 2016; Baudin *et al.*, 2017; Kim *et al.*, 2018). Further evidence supports that the host cell death subjected to ETI is controlled by the CC or TIR domain of NLR proteins in some cases (Bernoux *et al.*, 2011; Baudin *et al.*, 2017; Wang *et al.*, 2019b). However, there are other NLRs, including RPM1, Rx, Pm60, and Sr35, whose CC domains do not induce cell death in *N*. *benthamiana* (Hamel *et al.*, 2016; El Kasmi *et al.*, 2017; Zou *et al*., 2018; Bolus *et al.*, 2020), therefore we cannot generalize a signalling function for a particular CC or TIR domain.

Recognition of pathogen effectors results in the activation of NLR proteins and initiation of signalling (Takken *et al.*, 2006; Ade *et al.*, 2007; El Kasmi *et al.*, 2017; Praz *et al.*, 2017; Wang *et al.*, 2019b). In general, NLRs are present in an inactive state when there are no corresponding effectors (Kawano *et al.*, 2010; Stirnweis *et al.*, 2014; Baudin *et al.*, 2017; Praz *et al.*, 2017). Intramolecular interactions such as the interactions between the CC and NB domains and CC and LRR domains keep the NLR proteins in an autoinhibition state (Rairdan *et al.*, 2008; Wang *et al.*, 2015). To date, understanding of plant NLR activation has been relatively limited due to the lack of structural and biochemical mechanism data. Nevertheless, several studies have provided clues suggesting that oligomerization is crucial for plant NLR activation and signalling. For instance, self‐association has been observed in some CC‐NLR proteins, which are active when overexpressed (Ade *et al.*, 2007; Maekawa *et al.*, 2011; Cesari *et al.*, 2016). The crystal structure of ZAR1 shows that once activated, ZAR1 oligomerizes with other host proteins into an immune complex termed the ZAR1 resistosome, in which the oligomerization of the CC domains forms a funnel‐shaped structure that is necessary for the immune function of ZAR1 (Wang *et al.*, 2019b). Consistent with this result, evidence from several studies supports an important role of CC domain self‐association in cell‐death‐inducing activity (Maekawa *et al.*, 2011; Cesari *et al.*, 2016; El Kasmi *et al.*, 2017). Overall, these studies indicate that self‐association of plant NLRs might represent the activated state.

Because the NLR protein‐mediated immune response is often accompanied by the occurrence of cell death, cell death has been considered to be related to disease resistance in some cases (Dangl *et al.*, 2013; Cui *et al.*, 2015). Indeed, the exact relationship between cell‐death and disease‐resistance signalling is still ambiguous. Bai *et al. *(2012) proposed a bifurcation in cell‐death and resistance signalling based on the findings that nuclear‐localized MLA10 and cytosol‐localized MLA10 confer resistance and induce cell death, respectively. This conclusion is supported by other reports showing that the MLA10 CC domain mutant, L18E, is abolished in cell‐death activity, whereas the full‐length MLA10 harbouring the same mutation confers complete resistance against *Blumeria graminis* f. sp. *hordei* (Maekawa *et al.*, 2011). Nevertheless, Sr33, an ortholog of the MLA family, confers stem resistance only when it is located in the cytosol but not when it is confined to the nucleus (Cesari *et al.*, 2016). This result differs from MLA10 resistance signalling. In short, because experiment‐based evidence is considerably less, the correlation between cell death and disease resistance remains inconclusive.

Wheat powdery mildew is one of the most devastating diseases caused by *B. graminis* f. sp. *tritici* (Bgt). Recently, a powdery mildew resistance gene, *Pm21*, which confers broad‐spectrum resistance against Bgt, was cloned simultaneously by two groups (He *et al.*, 2018; Xing *et al.*, 2018). Their results showed that *Pm21* encodes a typical NB‐LRR protein with the CC domain in its N‐terminal. To better understand the mechanism of resistance mediated by *Pm21*, we conducted a functional analysis of the Pm21 protein, mainly focusing on the activity of the CC domain in cell‐death induction and the mechanism of the activity modulation. Our findings revealed significant roles of the CC domain in cell‐death induction, and also inhibition of the CC domain activity by intramolecular interactions. We propose a conception that Pm21‐mediated cell‐death and disease‐resistance signalling are potentially independent of each other.

## 2 RESULTS

### 2.1 Only the eCC domain of Pm21 has cell‐death‐inducing activity and self‐associates in planta

**FIGURE 1:**
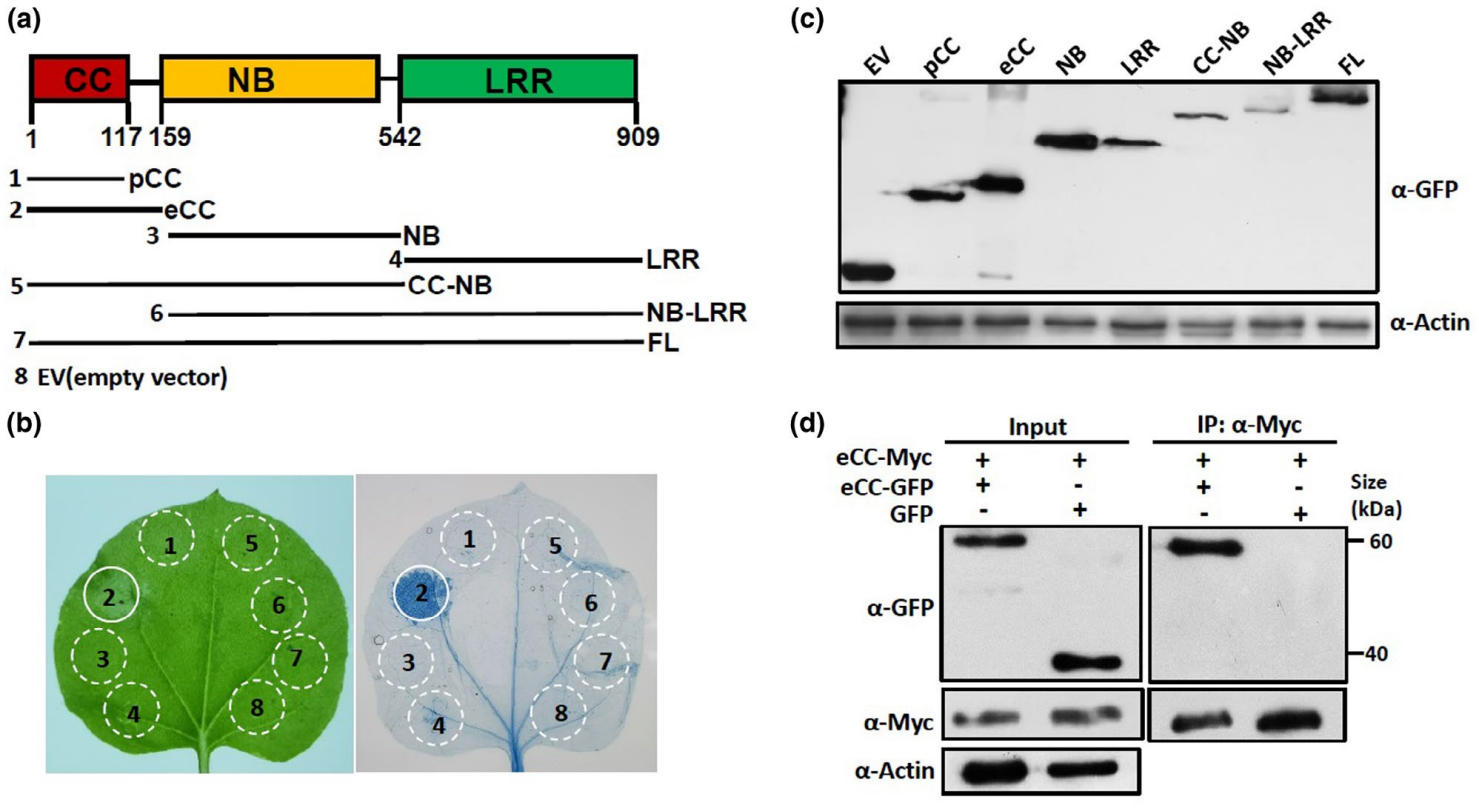
Only the eCC domain of Pm21 is capable of inducing cell death and self‐associates in planta. (a) Schematic diagram of the predicted Pm21 domain structure and the derived fragments expressed in *Nicotiana benthamiana*. Individual domains of Pm21 are represented by coloured boxes, and the relative positions of domains are indicated (upper panel). Pm21 fragments are drawn schematically in solid lines (lower panel). Empty vector (EV) was used as a negative control. (b) Analysis of cell‐death‐inducing activity. Indicated fragments fused with a C‐terminal green fluorescent protein (GFP) tag were transiently expressed in *N. benthamiana* leaves by *Agrobacterium tumefaciens* infiltration (agroinfiltration). Detached leaves were photographed at 40 hr post‐infiltration (hpi) (left), followed by trypan blue staining for cell‐death assay (right). Solid circles indicate cell death; dotted circles indicate no obvious cell death. The numbers represent the fragments shown in (a). The experiments were repeated twice with the same results. (c) Protein expression levels of Pm21 fragments shown by western blot. Total proteins were extracted from *N. benthamiana* leaves at 20 hpi and the tagged proteins were detected by western blot using an anti‐GFP antibody (α‐GFP). Equal protein loading is shown by immunoblot detection of actin (α‐Actin) throughout this article. (d) Analysis of self‐association of eCC fragment in planta. The eCC fragment fused to GFP or Myc was transiently expressed in the indicated combinations (+, agroinfiltrated construct; –, non‐agroinfiltrated construct) in *N. benthamiana*. Total proteins were extracted at 20 hpi and detected by western blot with anti‐GFP (α‐GFP) and anti‐Myc (α‐Myc) antibodies (Input). Coimmunoprecipitation was carried out with anti‐Myc antibody, and the proteins were detected by western blot with anti‐GFP and anti‐Myc antibodies. The same results were obtained in three independent experiments

Using structure prediction software, we identified the CC, NB, and LRR domains in Pm21 (Figure 1a). To investigate whether Pm21 can trigger cell death and determine the region crucial for cell‐death induction, we tested a series of Pm21 fragments, corresponding to different single or combined domains. These fragments were fused with the green fluorescent protein (GFP) tag at the C‐terminus and were transiently overexpressed in *N. benthamiana*. As shown in Figure 1b, only the extended CC (eCC, amino acid residues 1–159) domain corresponding to MLA10 residues 1–160, but not the predicted CC (pCC) domain (residues 1–117), induced a strong cell‐death response. This indicates that the Pm21 1–117 fragment might be a truncated CC domain and that the 118–159 region is crucial for the cell‐death‐inducing activity. We did not observe cell death after the expression of any of the other domains, that is, the NB and LRR domains (Figure 1b). Interestingly, the CC‐containing fragment CC‐NB and full‐length (FL) Pm21 did not cause cell death when overexpressed in *N. benthamiana.* These findings demonstrate that only the eCC domain has cell‐death‐inducing activity. Western blotting showed that all fusion proteins were properly expressed (Figure 1c). To confirm that the C‐terminal tag does not block protein functions, the Myc tag was fused to the C‐terminus of each fragment mentioned above. The transient overexpression assay showed that the cell‐death activity was not affected by different tags (Figure S1).

CC domain self‐association has been shown to be essential for function of NLRs (Maekawa *et al*., 2011; Cesari *et al.*, 2016; El Kasmi *et al.*, 2017). Thus, we wanted to know whether the Pm21 eCC domain also self‐associates in planta. We performed a coimmunoprecipitation (Co‐IP) experiment in *N. benthamiana*. The eCC domains fused to the GFP or Myc tag were transiently coexpressed in *N. benthamiana.* All proteins were verified to be properly expressed by immunoblotting. We found that the GFP‐fused eCC fragment coprecipitated with the Myc‐fused eCC fragment after immunoprecipitation with anti‐Myc beads. However, the entire GFP protein, which was used as the negative control, was not coprecipitated with the Myc‐fused eCC domain (Figure 1d). These results suggest that the active eCC domain of Pm21 self‐associates in planta. We also tested whether the pCC_1‐117_ fragment self‐associates in *N. benthamiana*. The Co‐IP experiment showed that the inactive pCC_1‐117_ fragment does not self‐associate in planta (Figure S2). We speculate that the region from 118 to 159 residues of Pm21 is probably crucial for self‐association.

### 2.2 The CC domain interacts with the NB and LRR domains in planta

Although the eCC domain has cell‐death signalling activity, the eCC‐containing fragments, such as CC–NB and CC–NB–LRR (FL Pm21), do not induce any cell‐death response when transiently overexpressed in *N. benthamiana*. This suggests that the eCC activity of cell‐death induction is negatively regulated by other domains in cis (when the two domains are fused in the same molecule). Notably, given that the overexpression of FL Pm21 protein does not trigger cell death, it is certain that the Pm21 protein is inactive in planta in the absence of pathogens.

**FIGURE 2:**
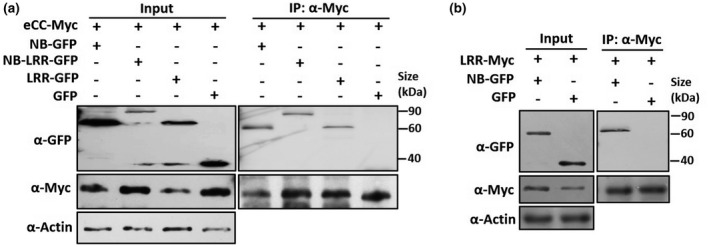
Interactions of different domains of Pm21 in planta. (a) The eCC fragment fused to Myc tag and other fragments fused to green fluorescent protein (GFP) tag were transiently expressed in *Nicotiana benthamiana* leaves in the indicated combinations (+, agroinfiltrated construct; −, non–agroinfiltrated construct). Total proteins were extracted at 20 hr post‐infiltration. The tagged proteins were detected by western blot with anti‐GFP and anti‐Myc antibodies (Input). Coimmunoprecipitation (IP) was carried out with anti‐Myc antibody, and the proteins were detected by western blot with anti‐GFP and anti‐Myc antibodies. Three independent experiments gave the same results. (b) The LRR domain fused to Myc tag and the NB domain fused to GFP or individual GFP were transiently expressed in *N. benthamiana* leaves in the indicated combinations. Samples were processed as described in (a). The experiments were repeated twice with the same results

To understand how the eCC activity is inhibited by other domains, we performed Co‐IP to test whether there is an interaction between the eCC domain with the NB domain or LRR domain. For this purpose, pairwise combinations of different domains fused with Myc‐tag and GFP‐tag were transiently coexpressed in *N. benthamiana*. Immunoblotting showed that all fused proteins were properly expressed in *N. benthamiana* (Figure 2a). The Co‐IP results showed that the eCC domain interacts with the NB, NBS–LRR, and LRR domains in vivo (Figure 2a). Unexpectedly, we observed that the NB domain interacts with the LRR domain (Figure 2b). We could not explain whether the NB–LRR interaction has an effect on the CC activity of cell‐death induction. From these interaction data, we hypothesize that the eCC domain interacts with the NB and LRR domains within Pm21 to inhibit the CC activity of cell‐death induction, thus maintaining the CC‐NB fragment and FL Pm21 protein in an autoinhibition state.

### 2.3 Mutated D491V in the MHD motif autoactivates Pm21, which is dependent on the LRR domain

**FIGURE 3:**
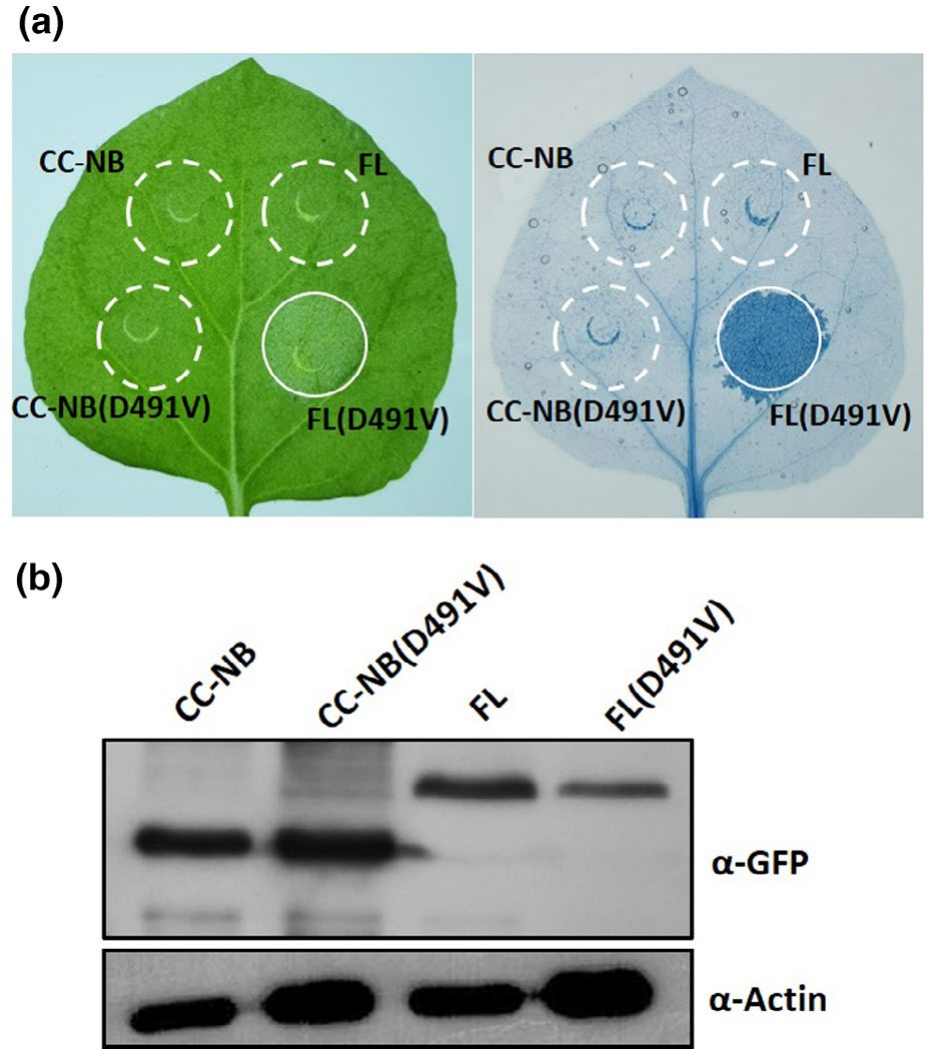
Analysis of cell‐death‐inducing activity of Pm21 D491V variants in *Nicotiana benthamiana.* (a) Green fluorescent protein (GFP)‐tagged Pm21 CC‐NB fragment or full‐length (FL) protein containing the D491V mutation were expressed in *N. benthamiana* leaves by agroinfiltration. Cell death was assessed by trypan blue staining at 40 hr post‐infiltration (hpi). The same results were obtained in three independent experiments. (b) Protein expression levels of each variant shown by western blot. Total proteins were extracted from *N. benthamiana* leaves at 20 hpi and detection was done by western blot with anti‐GFP antibody

The MHD motif in the NB domain is highly conserved among plant NLRs and mutations in the MHD motif result in the constitutive activation on transient expression in *N. benthamiana* leaves (Bendahmane *et al.*, 2002; Tameling *et al.*, 2006; Kawano *et al.*, 2010; Maekawa *et al.*, 2011; Wang *et al.*, 2015, 2018). To explore the effect of the MHD motif on the eCC activity, we introduced an aspartate‐to‐valine substitution in the MHD motif of the CC‐NB fragment and FL Pm21 protein, generating two variants CC‐NB (D491V) and FL (D491V) fused with the GFP tag in the C terminus. Transient overexpression of FL (D491V) in *N. benthamiana* induced a strong cell‐death response (Figure 3a), indicating that FL (D491V) is autoactive in the absence of pathogens. Surprisingly, we did not observe any cell death when the variant CC‐NB (D491V) was transiently overexpressed in *N. benthamiana*, suggesting that CC‐NB (D491V) still maintains an autoinhibition state. Immunoblotting showed that all fusion proteins were properly expressed at the expected size in *N. benthamiana* (Figure 3b). These data indicate that the residue D491 in the MHD motif is essential for autoinhibition of FL Pm21 and that the mutation of this residue results in constitutive activation. It is notable that the autoactivation of Pm21 mediated by the mutation of D491 requires the involvement of the LRR domain, inferring that LRR has a crucial role in Pm21 autoactivation.

### 2.4 Some mutations in the CC domain that lead to loss of *Pm21* resistance impair the cell‐death activity of the CC

In a previous study, we identified several dozen ethyl methanesulfonate‐induced mutations in *Pm21* that abolish *Pm21*‐confered powdery mildew resistance, 10 of which exist in the CC domain (He *et al.*, 2018). This prompted us to analyse whether mutations in the CC domain affect the cell‐death‐inducing activity of Pm21. First, we tested the 10 mutants for their responses to powdery mildew. The results revealed that, compared with the wheat‐resistant cultivar Yangmai 18, the 10 mutants showed phenotypes sensitive to Bgt (Figure S3). These data suggest that some specific residues in the CC domain are essential for *Pm21* resistance function.

**FIGURE 4:**
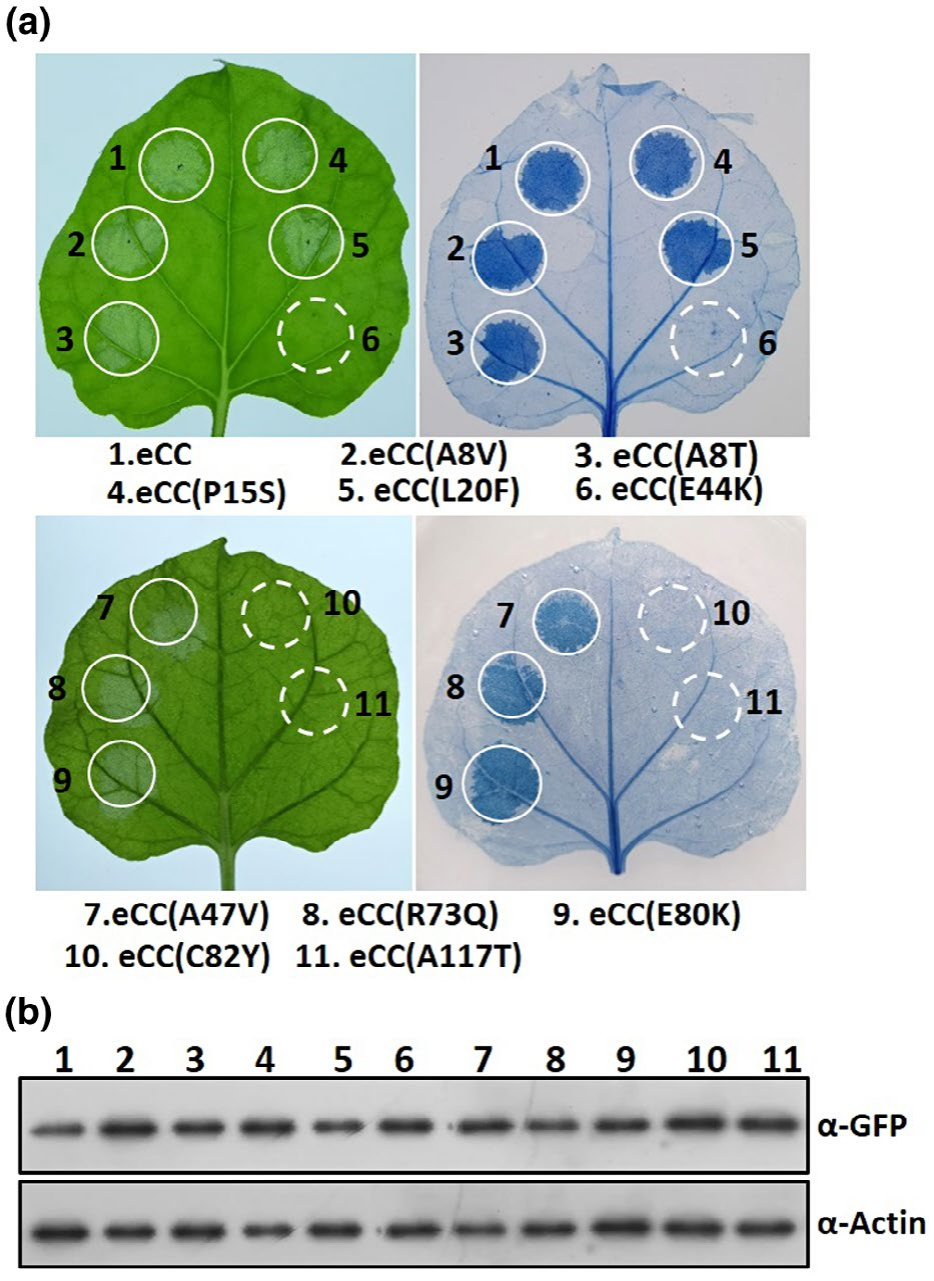
Cell‐death activity analysis of the Pm21 CC mutant variants in *Nicotiana benthamiana.* (a) Wild‐type and mutated constructs of Pm21 eCC domain fused to green fluorescent protein (GFP) tag were expressed in *N. benthamiana* leaves. Detached leaves were photographed at 40 hr post‐infiltration (hpi), followed by trypan blue staining for cell‐death assay. The experiments were repeated twice with the same results. (b) Protein expression levels of each CC variant shown by western blot. Total proteins were extracted from *N. benthamiana* leaves at 20 hpi and detection was done by western blot with anti‐GFP antibody

Next, we transiently overexpressed the 10 mutants and tested the cell‐death‐inducing activity in *N. benthamiana*. Considering that the wild‐type eCC domain is sufficient to induce a cell‐death response, we generated 10 eCC variants, each harbouring a single point mutation. Expression of all the variants was verified by immunoblotting (Figure 4b). The cell‐death assay showed that three variants, namely, E44K, C82Y, and A117T, completely lacked the ability to cause cell death, indicating that these three residues are crucial for the cell‐death‐inducing activity of the eCC domain (Figure 4a). However, seven variants, A8V, A8T, P15S, L20F, A47V, R73Q, and E80K, retained cell‐death‐inducing activity similar to the wild‐type eCC domain (Figure 4a), suggesting that these mutations do not alter the CC activity of cell‐death induction.

Structural exploration can provide insights into understanding the functions of plant NLRs. Homology modelling based on the structure of the CC domains of Sr33 and ZAR1, respectively, predicted that some residues mentioned above are located in the helices, which potentially contribute to the structure stability and interactions with other domains (Figure S4). Notably, in the Pm21 CC structure simulated according the ZAR1 CC, E44 and R73 form two hydrogen bonds with adjacent residues K124 and E56, respectively (Figure S4). The structural modelling results imply that these residues may have significance for the structure of Pm21.

### 2.5 D491V‐mediated Pm21 autoactivation is affected by mutations in residues R73 and E80

**FIGURE 5:**
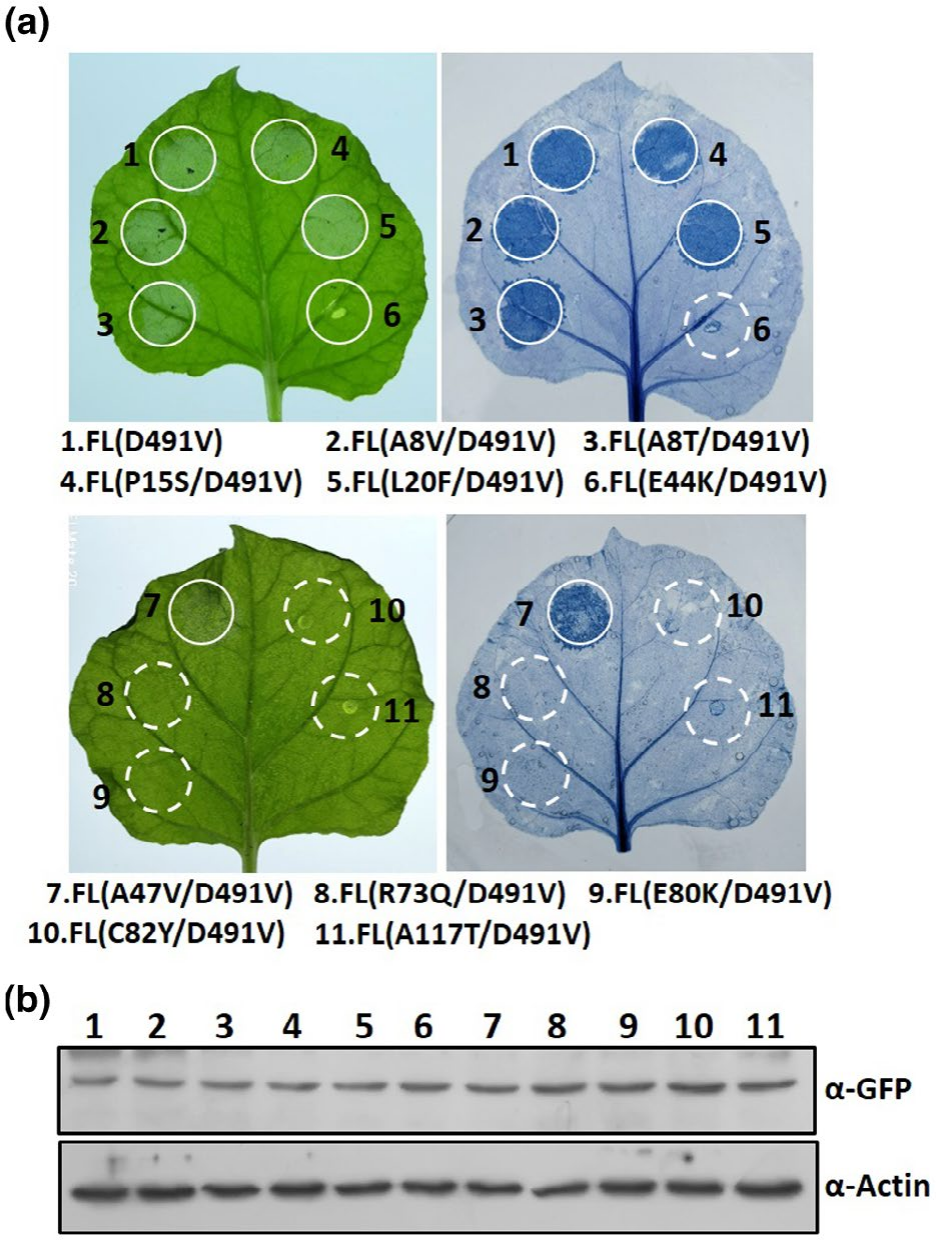
Analysis of cell‐death activity of the Pm21 full‐length (FL) variants harbouring the autoactivation mutation (D491V) and the CC mutations. (a) Green fluorescent protein (GFP)‐tagged Pm21 FL variants harbouring the autoactivation mutation (D491V) and the CC mutations were expressed in *Nicotiana benthamiana* leaves. Cell death was assayed by trypan blue staining at 40 hr post‐infiltration (hpi). The same results were obtained in three independent experiments. (b) Protein expression of the indicated variants. Total proteins were extracted from *N. benthamiana* leaves at 20 hpi and detection was done by western blot with anti‐GFP antibody

To further assess the effect of the 10 individual mutations on FL Pm21 activity, we introduced each mutation into the autoactive mutant FL Pm21 (D491V), generating 10 double mutation variants. When overexpressed in *N. benthamiana*, five variants, D491V + E44K, D491V + R73Q, D491V + E80K, D491V + C82Y, and D491V + A117T, triggered no or very weak cell death, whereas the other variants showed a cell‐death response similar to the autoactive variant D491V (Figure 5a). Immunoblotting detection demonstrated all variants proteins were expressed at similar levels (Figure 5b). Out of the five double mutation variants, D491V + E44K, D491V + C82Y, and D491V + A117T revealed the same phenotype as the corresponding eCC variants, further confirming that the residues E44, C82, and A117 are solely crucial for the CC activity. Notably, although mutations of residues R73 and E80 did not affect the cell‐death‐inducing activity of CC domain (Figure 4a), the same mutations suppressed the D491V‐mediated cell‐death activity of FL Pm21 (Figure 5a). Thus, these data suggest that mutations of R73 and E80 disrupt the function of Pm21 protein, but do not affect the inherent signalling properties of the individual CC domain. We conclude that the residues R73 and E80 may participate in autoactivation mechanism in the absence of pathogens.

In summary, some specific mutations in the CC domain disrupt Pm21 activity through either causing loss of the CC activity or interfering with the autoactivation that mimics activation of NLRs in the presence of corresponding effectors. In addition, as some mutations that abolish resistance to powdery mildew do not affect the cell‐death‐inducing activity, it is suggested that Pm21‐governed cell‐death signalling and disease‐resistance signalling are potentially independent of each other.

## 3 DISCUSSION

### 3.1 Roles of the CC domain of Pm21

In this study, we showed that that overexpression of Pm21 eCC_1‐159_, but not pCC_1‐117_, induces cell death in *N. benthamiana*. In fact, pCC and eCC correspond MLA10 1–120 and 1–160, respectively (Cesari *et al.*, 2016). This result is consistent with that of previous studies, which revealed that the full CC domains of MLA10, Sr33, and Sr50 are active in cell‐death signalling, whereas their truncated CC domains are not (Casey *et al.*, 2016; Cesari *et al.*, 2016). Our results substantiate the notion that individual CC domains of some plant NLRs have cell‐death‐inducing activity. However, this conclusion does not apply to all NLR proteins. For example, overexpression of the CC domains of Pm60 and Sr35 did not induce cell death (Zou *et al.*, 2018; Bolus *et al.*, 2020), suggesting that the CC domain of the NLR proteins might have other unknown functions. Unexpectedly, overexpression of either Pm21 CC‐NB or FL Pm21 did not trigger cell death, which is different from several reported NLR proteins, including MLA10, RGA4, Sr33, and Sr50, in which either the CC‐NB fragment or the FL protein has cell‐death‐inducing activity in the absence of effectors (Bai *et al.*, 2012; Cesari *et al.*, 2014, 2016; Bolus *et al.*, 2020). It is still unclear whether CC‐NLR proteins with similar structures have the same mechanisms of cell‐death induction.

In addition to implication in cell‐death signalling, the CC domain was shown to be important for *Pm21* resistance function. Similarly, mutations of the ZAR1 CC domain reduced disease resistance, indicating that the CC domain is critical for ZAR1 immune functions (Baudin *et al.*, 2017; Wang *et al.*, 2019b). *RPM1* resistance was affected by mutations in several residues of the CC domain (El Kasmi *et al.*, 2017). These data provided direct genetic evidence that an intact CC domain is required for NLR resistance function.

Interestingly, mutations of some residues in the CC domain do not affect CC‐induced cell death, but do suppress D491V‐mediated FL Pm21 autoactivation (Figure 5a), suggesting that the CC domain also participates in autoregulation of Pm21 in the absence of pathogen. This result agrees with the finding that mutations in the αC helix and the BB loop of the L6 protein lead to the loss of effector‐independent cell‐death induction without affecting L6 TIR domain self‐association (Bernoux *et al.*, 2011, 2016). Because of a lack of crystal structure, we cannot explain the mechanism by which the residues are involved in autoregulation of FL Pm21. We speculate that these residues might contribute to stabilization of autoactivation conformation.

### 3.2 Significance of intramolecular interactions

The results presented in this study suggest that the eCC domain of Pm21 self‐associates in planta, which agrees with previous reports on MLA10, Sr33, Sr50, and RPM1 (Maekawa *et al.*, 2011; Cesari *et al.*, 2016; El Kasmi *et al.*, 2017). In support of self‐association of the active CC domain, the crystal structure of activated ZAR1 shows that oligomerization of the CC domain is crucial for cell‐death activity (Wang *et al.*, 2019b). We also found that the pCC_1‐117_ fragment that has no cell‐death activity does not self‐associate in planta, further revealing a clear correlation between self‐association and cell‐death‐inducing activity. Building on these data, we believe that self‐association of the CC domain is required for cell‐death signalling.

The observation that overexpression of the Pm21 eCC domain, but not the CC‐NBS fragment and FL protein, induces cell death suggests that the CC‐NB fragment and FL protein are inactive (termed as autoinhibition) in planta in the absence of pathogens. Similar results have been reported for other NLR proteins (Kawano *et al.*, 2010; Stirnweis *et al.*, 2014; Baudin *et al.*, 2017; Praz *et al.*, 2017). We speculate that the cell‐death‐inducing activity of the CC domain is inhibited by the NB or LRR domain when NLRs are in an inactive state. This conclusion raises a question of how the activity of the CC domain is blocked by other domains. The Co‐IP experiment showed that the CC domain interacts with both NB and LRR domains. This result is also consistent with that of previous studies, in which physical interactions among different domains in several plant NLR proteins were identified (Moffett *et al.*, 2002; Rairdan *et al.*, 2008; Wang *et al.*, 2015). The crystal structure of inactive ZAR1 reveals that the CC domain interacts with multiple domains within ZAR1. The author concluded that these intramolecular interactions stabilize the inactive conformation of ZAR1 (Wang *et al.*, 2019a). Our biochemical data confirmed the interaction of the CC domain with the NB and LRR domains, which explains the observation that the Pm21 CC activity is inhibited by other domains. In addition, we also found that the NB domain interacted with the LRR domain of Pm21. Consistent with this result, NB–LRR interaction exists in autoinhibitory ZAR1 (Wang *et al.*, 2019a). Although we do not know the roles of NB–LRR interaction, it is possible that this interaction contributes to the regulation of the Pm21 activity.

Collectively, our results provide biochemical insights into the autoinhibition mechanism of Pm21, offering a referable model for understanding of NLRs, which are autoinhibitory in the absence of the effector. However, for the NLRs that are autoactive when overexpressed in *N. benthamiana*, whether or not the intramolecular interactions exert activity regulation remains to be determined.

### 3.3 Relation between cell‐death and disease‐resistance signalling

Because NLRs confer resistance and simultaneously induce cell death, it is generally believed that NLR‐mediated cell‐death signalling is closely related to disease‐resistance signalling (Dangl *et al.*, 2013; Cui *et al.*, 2015). However, the precise link between cell‐death and disease‐resistance signalling is largely unknown because direct downstream signalling molecules of NLR proteins remain obscue. In this study, we showed that some mutations in the CC domain led to loss of Pm21 resistance, whereas the same mutations did not affect the cell‐death activity of Pm21. This result provides evidence that Pm21‐mediated cell‐death and disease‐resistance signalling are potentially independent of each other. Consistent with this observation, mutations in N protein, a tobacco NLR conferring resistance to tobacco mosaic virus, show normal cell‐death response but fail to inhibit virus, indicating that cell death triggered by the N protein is not implicated in resistance to the virus (Dinesh‐Kumar *et al.*, 2000). Recently, *Arabidopsis* TNL (RRS1S‐RPS4)‐mediated cell death and bacterial resistance, which are both dependent on EDS1, have been shown to be two different branches (Lapin *et al.*, 2019). In addition, MLA10 signalling, taking place in the nucleus and cytoplasm, reveals the independence of cell‐death signalling and resistance signalling, respectively (Bai *et al.*, 2012). In short, these findings suggest that in some cases NLR‐induced cell death and disease resistance are probably not coupled.

Although current data reveal that Pm21‐mediated cell‐death and resistance signalling are independent of each other, we cannot answer whether this resistance is dependent on the occurrence of cell death, as we have no mutant that retains resistance but does not induce cell death. This work will provide a foundation for further studies of the relation between cell‐death and disease‐resistance signalling.

## 4 EXPERIMENTAL PROCEDURES

### 4.1 Plant materials

Wild‐type *N. benthamiana* plants and wheat plants were grown in a growth chamber at 23 °C and 70% relative humidity with a 16‐hr light period. The powdery mildew resistant wheat variety Yangmai 18 carrying *Pm21* and the susceptible variety Yangmai 9 were provided by the Yangzhou Academy of Agricultural Sciences (YAAS).

### 4.2 Evaluation of powdery mildew resistance

Wheat seedlings at the one‐leaf stage were inoculated with a predominant race of Bgt collected from Yangzhou region, China. Powdery mildew responses were evaluated at 7 days after Bgt inoculation. Because Yangmai 18 is immune to the Bgt isolate, the responses to powdery mildew are easily divided into two types, resistant and susceptible.

### 4.3 RNA extraction and cDNA synthesis

Extraction of total RNA from wheat seedlings leaves and synthesis of first‐strand cDNA were performed as described by Gao *et al. *(2016). The cDNAs were then used as templates for PCR amplifications.

### 4.4 Plasmid construction

The coding sequences of target proteins were amplified from cDNAs and inserted into pS1300‐GFP‐nos or pS1300‐Myc‐nos vector to create fusions with GFP or Myc tags on the C terminal, respectively. The target genes are driven by a constitutive promoter (Dong *et al.*, 2018). The point mutation vectors were generated by QuikChange site‐directed mutagenesis kit (Agilent Technologies). Constructs were confirmed by DNA sequencing. In this study, all PCR products used for cloning were generated using PrimeStar DNA polymerase (Takara). Cloning was performed by restriction‐ligation. Primers were purchased from YunYa Technologies (Zhengzhou, China), and are listed in Table S1.

### 4.5 *Agrobacterium*‐mediated transient expression

*Agrobacterium*‐mediated transient expression (agroinfiltration) was performed as described by Liu *et al.* (2010) with minor modifications. Briefly, *Agrobacterium tumefaciens* GV3101 harbouring the indicated vector constructs were grown at 28 °C in Luria Bertani‐man medium (0.5% yeast extract, 0.25% NaCl, 1% tryptone, 1% mannitol). The cultures were harvested by centrifugation and suspended in infiltration buffer (2% sucrose, 0.5% Murashige & Skoog basal salts, 10 mM 2‐(*N*‐morpholine)‐ethanesulfonic acid [pH 5.6], and 200 μM acetosyringone). The suspensions were diluted to OD_600_ of 0.8 using the same infiltration buffer. The solution was incubated at room temperature for 1–3 hr before infiltration. About 4‐week‐old *N. benthamiana* leaves were infiltrated. Each strain was infiltrated on three leaves from different plants. After infiltration, plants were kept in growth chambers under the same growth conditions.

### 4.6 Cell death assay in *N. benthamiana*

Cell‐death assay was performed as described by Bai *et al. *(2012). In short, leaves were photographed at 40 hr post‐infiltration (hpi). Detached leaves were soaked in trypan blue solution (10 ml lactic acid, 10 ml glycerol, 10 g phenol, 10 mg trypan blue, 50 ml ethanol, dissolved in 30 ml distilled water), and boiled for 5 min. Subsequently, leaves were destained in 2.5 g/ml chloral hydrate solution until the background was invisible.

### 4.7 Protein extraction, immunoblot, and Co‐IP assays

*A. tumefaciens* GV3101 carrying constructs expressing GFP‐ or Myc‐fused proteins were equally mixed and then infiltrated into *N. benthamiana* leaves according to the method described above. Protein isolation and related immunoblotting were performed as previously described with the minor modifications (Gao *et al.*, 2014). In brief, 0.2 g of *N. benthamiana* leaves were collected before cell death occurred (about at 20 hpi), ground in liquid nitrogen, then homogenized in 600 μl of plant extraction buffer (CWBIO). Extracts were incubated on ice for 30 min, followed by centrifugation at 12,000 × g for 20 min. The obtained supernatants were used for subsequent analysis. Fifty microlitres of supernatant was saved as input samples before Co‐IP, and the remaining supernatants was incubated with 4 μg of anti‐Myc monoclonal antibody (Proteintech) at 4 °C for 2–4 hr. Antiprotein complexes were captured by incubation with 18 μl of agarose resin conjugated with protein A/G (Proteintech) for 2–4 hr at 4 °C. After washing six times with IP buffer (50 mM HEPES [pH 7.5], 150 mM NaCl, 10 mM EDTA [pH 8.0], 0.2% Triton 100, 4 mM dithiothreitol and 1 × plant protein protease inhibitor mixture [Sigma‐Aldrich]), the immunocomplexes were heated at 95 °C for 5 min in 50 μl of 1 × SDS‐loading buffer and eluted by centrifugation. Immunoprecipitated proteins were analysed by western blot with the indicated antibodies together with input samples.

### 4.8 Homology structure modelling

Homology modelling of the Pm21 CC domain was performed with SWISS‐MODEL based on the structure of Sr33 (PDB: 2ncg) and Arabidopsis ZAR1 (PDB: 6j5w), respectively. The three‐dimensional structure and hydrogen bonds were mapped using PyMOL (http://www.pymol.org/).

## Supporting information


**FIGURE S1** Analysis of cell‐death‐inducing activity of Myc‐tagged fragments. All fragments fused with a C‐terminal Myc tag were transiently expressed in *Nicotiana benthamiana* leaves by *Agrobacterium tumefaciens* infiltration (agroinfiltration). Detached leaves were photographed at 40 hr post‐inoculation (left), followed by trypan blue staining for cell‐death assay (right). Solid circles indicate cell death; dotted circles indicate no obvious cell death. Empty vector (EV) was included as a negative control. The experiments were repeated twice with the same results

**FIGURE S2** Investigation of self‐association of the pCC domain in planta*.* The predicted CC (pCC) fragment fused with the C‐terminal GFP or Myc tag was transiently expressed in *Nicotiana benthamiana*. Total proteins were extracted at 20 hr post‐inoculation and detected by western blot with anti‐GFP and anti‐Myc antibodies (Input). Coimmunoprecipitation was carried out with anti‐Myc antibody, and the proteins were detected by western blot with anti‐GFP and anti‐Myc antibodies. Equal protein loading in the input is shown by detection of actin

**FIGURE S3** Responses of mutants containing the Pm21 CC domain mutations to *Blumeria graminis* f. sp. *tritici*. Wheat seedlings at the one‐leaf stage were inoculated with a predominant race of *B. graminis* f. sp. *tritici*. Powdery mildew responses were evaluated at 7 days after inoculation. Yangmai 18 and Yangmai 9 were used as the resistant and susceptible controls, respectively

**FIGURE S4** Homology modelling and structural superposition. The structures of Pm21 CC_11‐117_ (left, wheat) and CC_12‐140 _(right, cyan) were simulated based on the structures of Sr33 CC (left, cyan) and ZAR1 CC (right, cyan), respectively. Residues are shown in stick representation. Hydrogen bonds are shown in red boxes with yellow dotted lines

**TABLE S1** Primers list

## Data Availability

The sequence of *Pm21* can be found in GenBank at https://www.ncbi.nlm.nih.gov/nuccore with under accession number MF370199. The other data that support the findings of this study are available from the corresponding author upon reasonable request.
