# Supplementary figures and images for: Pm21 CC domain activity modulated by intramolecular interactions is implicated in cell death and disease resistance
Source: Mol Plant Pathol. 2020 May 18;21(7):975–84. doi: 10.1111/mpp.12943 (PMC7279971; doi:10.1111/mpp.12943)

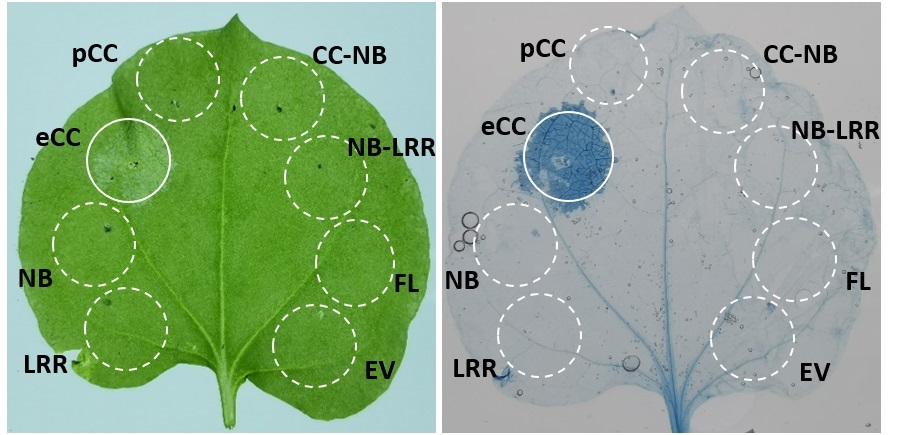

Supplement: Supplementary file 1 — FIGURE S1 Analysis of cell‐death‐inducing activity of Myc‐tagged fragments. All fragments fused with a C‐terminal Myc tag were transiently expressed in Nicotiana benthamiana leaves by Agrobacterium tumefaciens infiltration (agroinfiltration). Detached leaves were photographed at 40 hr post‐inoculation (left), followed by trypan blue staining for cell‐death assay (right). Solid circles indicate cell death; dotted circles indicate no obvious cell death. Empty vector (EV) was included as a negative control. The experiments were repeated twice with the same results [file MPP-21-975-s001.jpg]

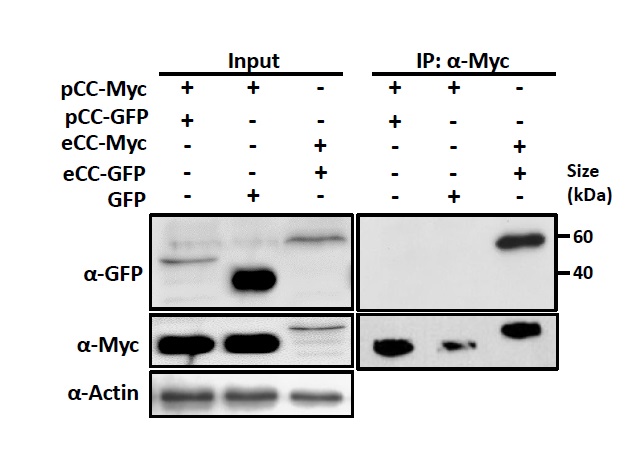

Supplement: Supplementary file 2 — FIGURE S2 Investigation of self‐association of the pCC domain in planta. The predicted CC (pCC) fragment fused with the C‐terminal GFP or Myc tag was transiently expressed in Nicotiana benthamiana. Total proteins were extracted at 20 hr post‐inoculation and detected by western blot with anti‐GFP and anti‐Myc antibodies (Input). Coimmunoprecipitation was carried out with anti‐Myc antibody, and the proteins were detected by western blot with anti‐GFP and anti‐Myc antibodies. Equal protein loading in the input is shown by detection of actin [file MPP-21-975-s002.jpg]

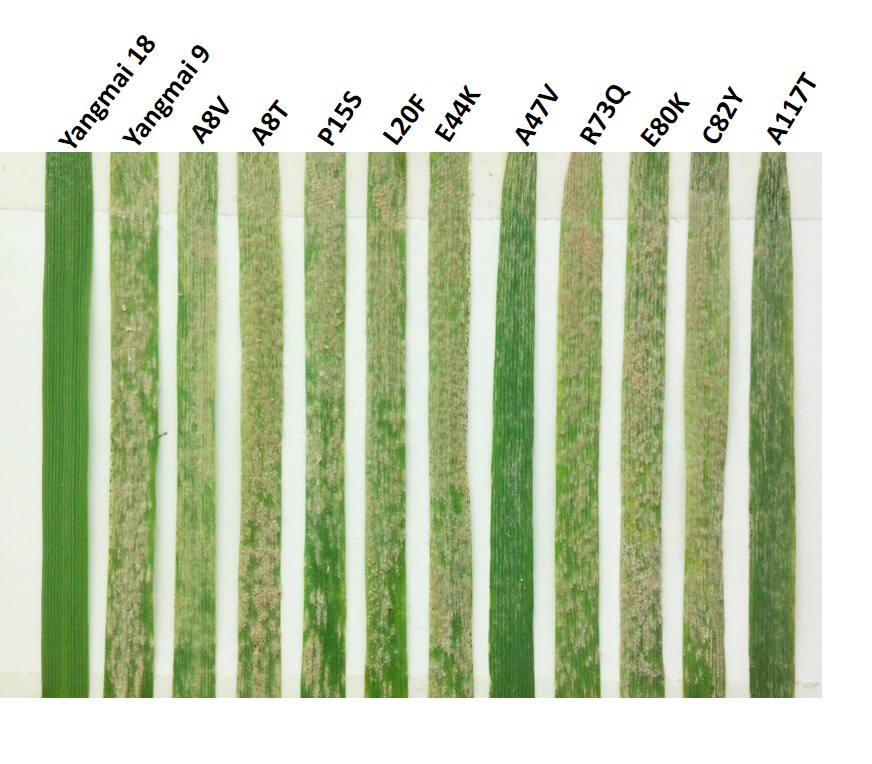

Supplement: Supplementary file 3 — FIGURE S3 Responses of mutants containing the Pm21 CC domain mutations to Blumeria graminis f. sp. tritici. Wheat seedlings at the one‐leaf stage were inoculated with a predominant race of B. graminis f. sp. tritici. Powdery mildew responses were evaluated at 7 days after inoculation. Yangmai 18 and Yangmai 9 were used as the resistant and susceptible controls, respectively [file MPP-21-975-s003.jpg]

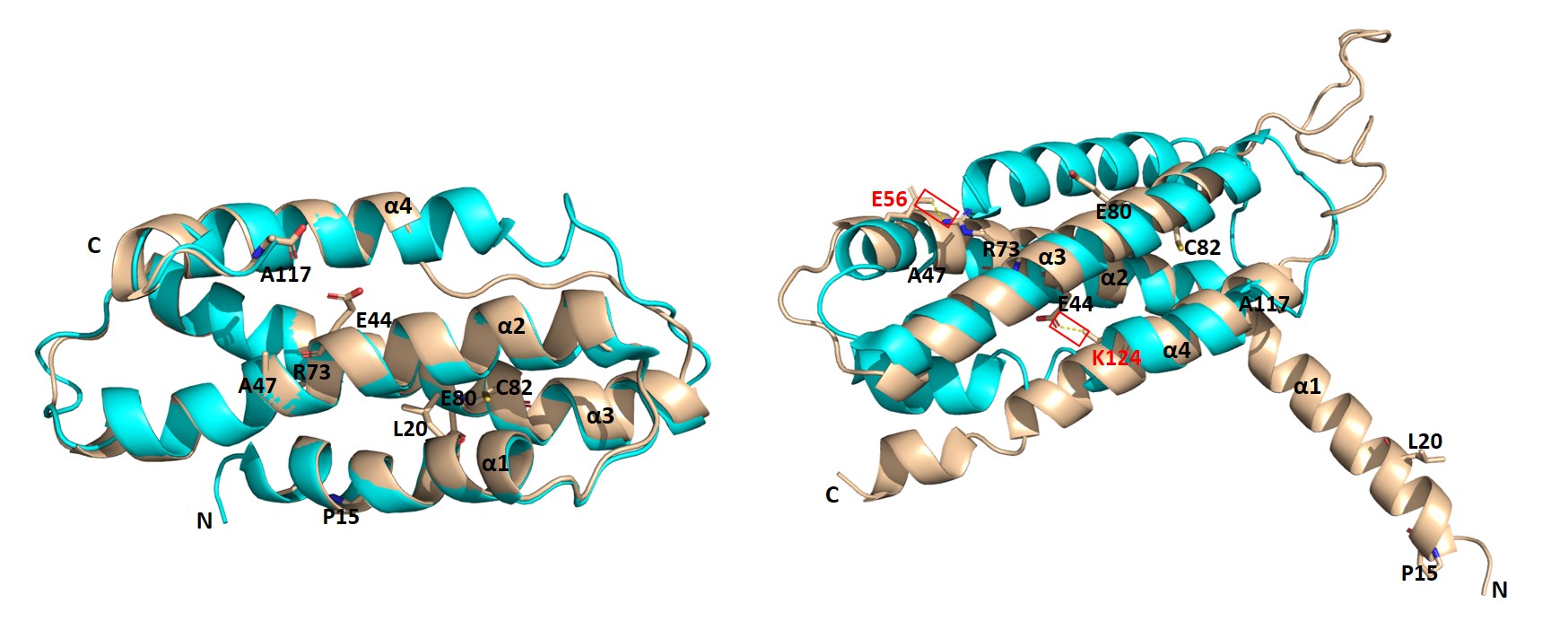

Supplement: Supplementary file 4 — FIGURE S4 Homology modelling and structural superposition. The structures of Pm21 CC11‐117 (left, wheat) and CC12‐140 (right, cyan) were simulated based on the structures of Sr33 CC (left, cyan) and ZAR1 CC (right, cyan), respectively. Residues are shown in stick representation. Hydrogen bonds are shown in red boxes with yellow dotted lines [file MPP-21-975-s004.jpg]
