# Supplementary material for: Pm21 CC domain activity modulated by intramolecular interactions is implicated in cell death and disease resistance
Source: Mol Plant Pathol. 2020 May 18;21(7):975–84. doi: 10.1111/mpp.12943 (PMC7279971; doi:10.1111/mpp.12943)
Supplement: Supplementary file 5 — TABLE S1 Primers list [file MPP-21-975-s005.docx]

| **Primers for the coding sequence PCR** | |
| --- | --- |
| **Primer name** | **Sequence(5’-3’)** |
| Pm21- F | TCACTGCAGATGTCTGCACCGGTCGTCAG |
| Pm21-CC_117_-R | TACACTAGTTTAAGCAATACGATGACGCT |
| Pm21-CC_159_-R | ACTACTAGTGATTGCCAACATCCGAGTAT |
| Pm21-NB_160_-F | CTACTGCAGTACAAGCAGGCAACGGGGCT |
| Pm21-NB_542_-R | TGAACTAGTGTTTTCTGATGTTGCATCGT |
| Pm21-LRR_543_-F | TAACTGCAGATTCATACTAGCATGCAACA |
| Pm21-R | TCTACTAGTAAGTAAAACTGGGACCACAT |

Table S1

Note: Sequences underlined indicate restriction sites
